# Online self-compassion-based interventions on patient outcomes in patients with cancer: a systematic review

**DOI:** 10.1007/s00520-026-10660-8

**Published:** 2026-04-23

**Authors:** Emine Çetiner, Selma Turan Kavradım

**Affiliations:** 1 Internal Medicine Nursing PhD Program, Institute of Health Sciences, Akdeniz University, Antalya, Türkiye; 2 Department of Internal Medicine Nursing, Faculty of Nursing, Akdeniz University, Antalya, Türkiye

**Keywords:** Cancer, Nursing, Online self-compassion-based interventions, Self-compassion, Systematic review

## Abstract

**Purpose:**

This systematic review investigates the effectiveness of online self-compassion-based interventions and their impact on the outcomes of cancer patients.

**Methods:**

A comprehensive search was carried out in the Web of Science Core Collection, Cochrane Library, CINAHL, PubMed, ScienceDirect, Scopus, Ovid, and SpringerLink databases without any year limitation until January 01, 2026. Cochrane and PRISMA guidelines were used for systematic review and reporting. RoB2 and Joanna Briggs Institute guidelines were utilized to assess the risk of bias.

**Results:**

Eight studies included in the systematic review were conducted between 2017 and 2025 in Australia, the USA, the Netherlands, China, and Türkiye. The self-compassion interventions applied in the studies included mindful self-compassion, self-compassion-focused writing, compassion mind training, and kindness interventions. Online self-compassion-based interventions have been found to increase patients’ levels of self-compassion, mindfulness, body image appreciation, well-being, self-acceptance, posttraumatic growth, and positive affect while reduce their levels of self-criticism, social isolation, anxiety, stress, and depression, body image distress.

**Conclusions:**

Studies have shown the positive effects of self-compassion-based interventions on patient outcomes. A sensitivity analysis that included only randomized controlled trials demonstrated robustness in all outcomes except anxiety; however, some research results were obtained from a single randomized controlled trial. Since some study results were derived solely from quasi-experimental studies, they were not included in the analysis. Therefore, more randomized controlled research is required to integrate these interventions into clinical patient care. Online self-compassion interventions can contribute to the planning of holistic nursing interventions for patient outcomes.

**Supplementary Information:**

The online version contains supplementary material available at 10.1007/s00520-026-10660-8.

## Introduction

Cancer is the second leading cause of death worldwide after cardiovascular disease [1]. Based on World Health Organization data, the number of new cases worldwide in 2022 was calculated to be approximately 20 million, and the number of deaths was reported to be 10 million [2]. Diagnosis of cancer and stages of treatment affect the patients’ lives in many ways, and both physical and psychosocial problems may arise [3]. Self-compassion is being receptive to one’s feelings that cause pain and distress, approaching oneself with affectionate, compassionate attitudes, and being understanding and accepting negative encounters as a natural part of life [4, 5]. Self-compassion interventions, which are an effective emotion regulation mechanism for patients with cancer to cope with difficult situations [4], have been increasing, particularly in patients with cancer in recent years [6, 7]. In patients with cancer, self-compassion has been associated with lower anxiety, depressive symptoms [8], cancer-related fatigue [9], body image disturbances [10], body shame [10], and higher sleep quality [11], treatment adherence [12], well-being [13], and quality of life [14]. Self-compassion interventions can be implemented either face-to-face [15, 16] or online [6, 7]. It is reported that face-to-face self-compassion interventions for patients with cancer have certain limitations [17]. For this reason, the effects of online self-compassion interventions have been researched in recent years [18, 19]. Examining the effects of online self-compassion interventions on the health outcomes of patients with cancer is predicted to improve patient outcomes by contributing to health services.

### Background

Cancer, whose global burden is constantly increasing, affects patients in many physical and psychological ways [20]. Self-compassion is an effective emotion regulation mechanism and coping strategy [4] that involves developing a kindness, an understanding, and a less judgmental attitude toward oneself to help cancer patients cope with difficult situations [21]. Self-compassion consists of six subcomponents, of which the positive sub-dimensions of self-compassion are self-kindness, common humanity, and mindfulness, and the negative sub-dimensions are self-judgment, isolation, and over-identification [4, 5]. The sub-dimensions of self-compassion, self-kindness, common humanity, and mindfulness interact with each other and help patients cope with stressful life events and display a balanced and understanding attitude [22]. Among the psychological interventions applied to patients with cancer, self-compassion interventions have recently gained importance and include various methods that can affect the outcomes of patients [17]. Strengthening self-compassion helps increase subjective well-being, self-acceptance, and respect for the body [23].

A variety of self-compassion interventions can be implemented [7, 24–26]. These interventions began with self-compassion-focused writing developed by Leary and colleagues [27], and continued with mindful self-compassion developed by Neff and Germer [28]. In addition, the compassion-focused therapy approach developed by Gilbert is also used [29]. The literature also includes kindness interventions that handling various approaches, such as self-kindness practices, kindness to others practices, and self-compassion meditation [26]. Self-compassion interventions in patients with cancer increase resilience [30], facilitate individuals’ adaptation to the body changes, improve body image appreciation [31], reduce pain [30], stress [32], anxiety [30], and depression [30]. These interventions are reported to facilitate individuals’ adaptation to physical and psychosocial changes experienced during cancer treatment, improve their quality of life, and make it easier for them to accept and cope with negative life experiences [7, 33]. In addition to these, self-compassion interventions help patients with cancer accept their challenging life experiences, improve emotion regulation skills, and reduce feelings of isolation [17].

Self-compassion interventions can be delivered face-to-face [15, 16] and, in recent years, have also delivered online [6, 7]. Although face-to-face interventions are frequently applied for patients with cancer provide social support [34, 35], it is emphasized that face-to-face interventions have various limitations, such as transportation and access difficulties, high cost, confidentiality, privacy, and stigma concerns [17, 36]. Online interventions increase access to healthcare by removing barriers such as physical distance, mobility issues, and time constraints [37, 38]. Since they do not require the therapist’s constant presence, they enable individuals to access services more quickly and on time; their self-directed structure contributes to overcoming attitudinal barriers that limit participation in face-to-face interventions [39]. In addition, the fact that online interventions are perceived less as “therapy” can reduce resistance to seeking psychological support [40]. These features increase cost-effectiveness, support equal access to services, and make it possible to reach individuals who would not seek support under normal circumstances [37, 38]. Furthermore, the use of online coaching, self-monitoring tools, and other information and communication technologies strengthens participant engagement and adherence to interventions [41]. Current evidence shows that online psychological interventions can be as effective as face-to-face applications [42]. The effects of online self-compassion interventions on different populations have been discussed previously [18, 19]. However, no systematic review in the literature examining the impact of online self-compassion interventions on cancer patients’ outcomes. Evaluating the effectiveness and sustainability of online self-compassion interventions in cancer patients may contribute to improving patient outcomes and guiding future research.

## Method

### Purpose

This systematic review aims to investigate the effectiveness of online self-compassion-based interventions and their impact on the outcomes of cancer patients. This systematic review aims to clarify and provide answers to the identified research questions:

1. What are the methods used in online self-compassion-based interventions?
2. What is the intervention protocol (frequency, duration, follow-up) of online self-compassion-based interventions?
3. Are online self-compassion-based interventions effective on patients’ outcomes?

### Design

This systematic review was carried out in line with the systematic review methods outlined in the Cochrane Handbook and reported based on The Preferred Reporting Items for Systematic Reviews and Meta-Analyses (PRISMA) 2020. We registered with PROSPERO to conduct a systematic review on online self-compassion-based intervention in patients with cancer (PROSPERO Registration Number: CRD42024565875).

### Search strategy

Web of Science Core Collection, Cochrane Library, CINAHL, PubMed, Science Direct, Scopus, Ovid Total Access Connection, and SpringerLink databases were searched between July 2024 without a year limitation. The keywords used in the search were “tumor OR neoplasia OR cancer” AND “self-compassion” OR “self compassion” OR “mindful self-compassion” OR “self-kindness” OR “self-forgiveness” AND “internet OR web OR online OR videoconference” keywords and combinations (Appendix File, Table S1). The PRISMA guidelines were used to report this study.

### Eligibility criteria

The PICOS method was utilized to determine the inclusion and exclusion criteria. This method includes the population (P), intervention (I), comparison group (C), study results (O), and study design (S) [43]. The selection criteria for the study were as follows; (P)18 years and older who were undergoing or had undergone cancer treatment, (I) implemented self-compassion-based interventions with digital, online, web based, mobile, (C) compared with routine care or a different method, or evaluated the effects of online self-compassion-based interventions with one group, (O) psychological symptoms such as self-compassion and sub-dimensions, anxiety, stress, depression, body image outcomes, well-being and sub-dimensions, (S) Randomized controlled or quasi-experimental studies that included adult patients with cancer. In addition, only studies published in English have been included. The exclusion criteria for the study were as follows; studies that included children, partners, caregivers, family centered, interventions not centered on self-compassion such as mindfulness-based stress reduction, compassion-based interventions, delivered face to face, had study designs other than experimental studies (case reports, guidelines, retrospective and prospective cohort, retrospective descriptive, descriptive studies), whose full text could not be accessed, and were prepared as protocols, conference posters, oral abstract.

### Selection of studies

**Fig. 1:**
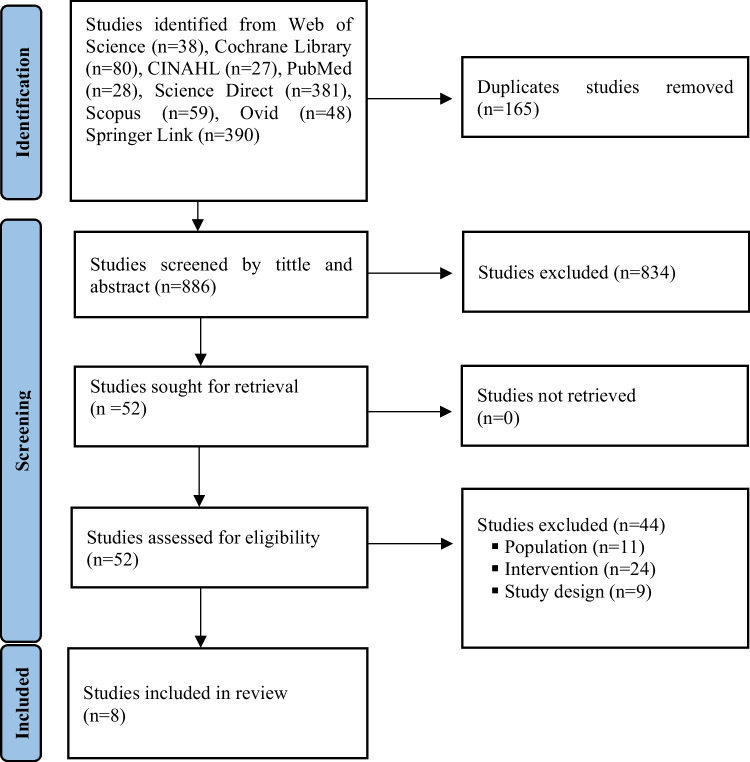
PRISMA flowchart for the selection of studies

In the database search, 1051 studies were reached by scanning Web of Science Core Collection (*n* = 38), Cochrane Library (*n* = 80), CINAHL (*n* = 27), PubMed (*n* = 28), Science Direct (*n* = 381), Scopus (*n* = 59), Ovid Total Access Connection (*n* = 48), and Springer Link (*n* = 390) databases on January 01, 2026. 165 (one hundred sixty-five) duplicate studies were extracted using the EndNote21 program. In the second step, the studies were evaluated for relevance to the title and abstract, and 834 studies that were not relevant were removed. The full text of 52 articles was evaluated by two independent authors (EÇ, STK), and a final consensus was reached regarding the inclusion and exclusion criteria. After full-text screening, 44 studies were excluded due to not meeting the inclusion criteria. As a result, eight studies were included in the systematic review, and the selection process was completed (Fig. 1, PRISMA).

### Methodological quality assessment of the studies

Two independent authors evaluated the included studies for risk of bias. The Cochrane risk-of-bias tool (RoB2), second version [43] was used for randomized studies. This risk tool systematically assesses the risk of bias by addressing the domains of (1) randomization process, (2) deviations from the intended intervention, (3) incomplete outcome data, (4) measurement bias of results, and (5) selective reporting, and overall bias. For non-randomized studies, the Joanna Briggs Institute (JBI) Quasi-Experimental Studies Checklist was used [44]. The JBI quasi-experimental studies assessment tool was used to identify the risk of bias in the design, conduct, and analysis of the study. The checklist for quasi-experimental studies has nine items. Each item was assessed as “yes,” “no,” “unclear,” or “not applicable.”

### Data abstraction and synthesis

A table was created for the articles to be included in the review. Relevant studies were evaluated in terms of author, year, country, study design, aim, details of group (type of cancer, age), intervention received, and control group description, online intervention categories, session detail, main outcome variable(s), and main findings. Meta-analyses were not conducted due to the diversity of online self-compassion interventions for patients with cancer, as well as the variation in the outcome parameters measured. Narrative analysis was used to report the results.

## Results

### Description of the included studies

Table 1 shows the description of the included studies in the systematic review. It was found that there were a total of seven studies, five randomized controlled studies, and three quasi-experimental studies. Studies conducted in Australia [25, 31], the USA [26, 45], the Netherlands [24, 46], China [7], and Türkiye [6]. It is seen that the use of online self-compassion interventions has increased over the years, and most studies (*n* = 2, 28.5%) belong to 2021 [25, 46] and 2023 [24, 26]. The most recent study was published in 2025 [6], while the first study was published in 2017 [45]. Sample sizes vary between 25 [45] and 304 [31]. In total, 838 cancer patients were included in five randomized controlled studies [6, 7, 25, 26, 31] and three quasi-experimental studies [24, 45, 46]. The cancer population consisted of people with newly diagnosed cancer, young adult cancer survivors, early-stage breast cancer survivors, head and neck cancer survivors, and breast cancer survivors. Breast cancer (*n* = 5, 62.5%) was examined more than other cancer types [6, 7, 25, 26, 31].

**Table 1:** Description of the included studies

Author, year/country	Study design	Aim	Detail of group (type of cancer, age)	Intervention received and control group description	Online intervention categories	Session detail	Main outcome variable(s)	Main findings
Austin et al., 2023, Netherlands	Pretest–posttest design	To evaluate the use, appreciation, and impact of the app	People with newly diagnosed cancer: breast cancer, colorectal or esophagus cancer, prostate, lymphoma, melanoma, ovarium, lung, other cancer (*n* = 71) Mean age: 52.5 ± 11.7	Compassionate mind training. Compassionate mind training includes psycho-education, imagery exercises, self-compassion-based expressive writing exercises, compassionate body scans, and loving-kindness meditation Instructor: Self-guided	Mobile application (Compas-Y) and audio	Each module 60–90 min, one module per week, a total of 6 modules, 8 weeks	Anxiety and depression Well-being Social isolation Cancer-related resilience Self-compassion Self-criticism Fears of giving and receiving compassion Cognitive coping and emotion regulation strategies	Statistically significant reduction in anxiety, in self-criticism (inadequate self and self-blame), but not self-compassion. Compas-Y supported patients in aspects of their mental health
Campo et al., 2017, USA	Pretest–posttest design	To assess the feasibility, acceptability, and technical challenges of a videoconference intervention for cancer survivors and examine changes in psychosocial outcomes	Young adult cancer survivors: lymphoma, thyroid, sarcoma, ovarian, breast, leukemia, central nervous system, lung, brain (*n* = 25) Mean age: 26.7 ± 2.0	Mindful self-compassion intervention. Intervention includes compassionate friend meditation, body scan, here and now stone, meditation, soften-soothe-allow meditation, and gratitude phone photos Instructor: Completed the mindful self-compassion teacher training program and had three years of mindfulness-based instructor experience	Videoconference tool (Cisco WebEx) and audio	Group-based 90-min. videoconference sessions held weekly over 8 weeks	Self-compassion Mindfulness Anxiety Depression Social isolation Body image distress Resilience Posttraumatic growth	All psychosocial outcomes, except for resilience, demonstrated significant changes. While anxiety, depression, social isolation, and body image distress decreased, self-compassion, posttraumatic growth, and mindfulness increased
Chen et al., 2024, China	A randomized controlled trial	To assess the effectiveness of a 6-week online Mindful Self-Compassion intervention to reduce the negative body image in breast cancer patients	Breast cancer patients (*n* = 64) IG: *n* = 32 CG: *n* = 32 Mean age: no information	Mindful self-compassion intervention. Intervention includes coping with difficult emotions and negative core beliefs, finding your compassionate inner voice, self-compassionate body scan, foot meditation and self-appreciation IG: mindful self-compassion CG: usual care Instructor: a clinical psychology professor and psychology graduate student with extensive professional experience in mindfulness and MSC	Videoconference tool (Tencent Meetings software) and audio	Total of a 6-week course 2.5 h of classes per week plus a half-day retreat Half-day retreat between weeks 4 and 5 for 4 h in silence while performing various meditations, restorative yoga, and joyous eating	Self-compassion Self-acceptance Perceived stress Body image distress	It was concluded that the online Mindful Self-Compassion program can improve self-compassion and self-acceptance and reduce perceived stress and body image distress
Çalışkan ve Kutlu, 2025, Türkiye	Randomized controlled trial	To evaluate the effects of mindfulness based self-compassion program on the ontological well-being of breast cancer survivors during the post-treatment phase	Breast cancer survivors (*n* = 71) IG: *n* = 35 CG: *n* = 36 Mean age: IG: 43.98 ± 6.0 CG: 45.15 ± 6.0	Mindful self-compassion intervention. Intervention includes mindful breathing and movement, present-moment awareness exercises, compassionate imagery, compassionate letter writing, peer sharing, and normalization of experiences, home assignments consisting of daily audio-guided practice and reflective journaling tasks IG: mindful self-compassion CG: usual care Instructor: by the first author, a certified psychiatric nurse trained in mindfulness-based interventions	Videoconference tool (Zoom) and audio	One session per week, 8 sessions in total, each session lasting approximately 2.5 h	Ontological well-being Ontological well-being sub-dimensions: Nothingness Hope Regret Take action	Mindfulness-based self-compassion increased patients' overall ontological well-being and the subdimensions of hope, action, and nothingness. No significant difference was observed in the regret subscale
Haydon et al., 2023, USA	Randomized controlled trial	To test the efficacy of kindness-focused practices among early-stage breast cancer survivors	Early-stage breast cancer survivors (*n* = 137) IG^1^: *n* = 34 IG^2^: *n* = 33 IG^3^: *n* = 32 CG: *n* = 34 Mean age: IG^1^: 63.09 ± 7.98 IG^2^: 62.52 ± 5.68 IG^3^: 62.59 ± 5.38 CG: 62.41 ± 6.39	Kindness interventions: kindness to others (kind, generous, or thoughtful acts directed towards others), kindness to self (kind acts directed towards themselves), self-kindness meditation (listen to a 5-min guided meditation) IG^1^: acts of kindness to others IG^2^: acts of kindness to self IG^3^: self-kindness meditation CG: daily-activity-writing control Instructor: an experienced mindfulness instructor (only expressed self-compassion meditation)	Online blog-publishing Platform (only expressed self-compassion meditation) No information is available regarding the presentation of other interventions	Online three interventions, three activities each week for 4 weeks	Psychological well-being Depressive symptoms Social support Self-kindness	No differences were found in the primary outcomes. Participants in the acts of kindness to others condition reported greater increases in social support, and participants in the self-kindness meditation condition reported greater decreases in self-kindness
Melissant et al., 2021, Netherlands	Pretest–posttest design	To investigate the reach and effects of My Changed Body, an expressive writing activity based on self-compassion	Head and neck cancer survivors (*n* = 87) Mean age: 66 ± 11.2	Self-compassion-focused writing intervention. Intervention involves writing about patients’ deepest thoughts and feelings regarding a negative event related to their changing bodies, using written prompts to increase self-compassion towards themselves and their post-cancer bodies. The intervention also includes participants practicing self- kindness, common humanity, and mindfulness Instructor: Self-guided	Booklet and web-based	30 min, for 1 month, single session	Body image-related distress Body appreciation Self-compassion Psychological distress Health-related quality of life Head and neck cancer symptoms (problems with wound healing and social contact) Sexual problems	Self-compassion improved significantly, and no significant effect on body image-related distress was found. No significant effects were observed on other secondary outcomes
Mifsud et al., 2021, Australia	Randomized controlled design	To assess the feasibility and acceptability of My Changed Body, with and without an additional meditation component, on body image distress and related psychological outcomes	Breast cancer survivors (*n* = 79) IG^1^: *n* = 39 IG^2^: *n* = 17 CG: *n* = 23 Mean age: IG^1^: 57.54 ± 13.64 IG^2^: 60.88 ± 8.42 CG: 57.39 ± 9.53	Self-compassion-focused writing intervention. Intervention includes being invited to write a compassionate letter to themselves, containing gentle advice to their bodies; to connect with others who may have had similar experiences; and to become mindful of their experiences and reactions IG^1^: a self-paced 6-step evidence-based writing intervention IG^2^: writing activities and listening to a brief 5-min self-compassion-based audio meditation CG: expressive writing with usual care Instructor: self-guided	Web-based and audio	6-step, 30 min, for 1 month, single session	Body image distress Body appreciation Self-compassion Positive and negative affect Depression, anxiety, and stress	Body image distress decreased across all conditions, self-compassion increased, and anxiety decreased for MyCB and meditation compared to MyCB and the expressive writing group
Sherman et al., 2018, Australia	Randomized controlled trial	To assess whether usual care plus My Changed Body can promote adjustment to bodily changes	Breast cancer survivors (*n* = 304) IG: *n* = 149 CG: *n* = 155 Mean age: IG: 57.50 ± 8.98 CG: 57.23 ± 9.97	Self-compassion-focused writing intervention. Intervention involves writing about patients’ deepest thoughts and feelings regarding a negative event related to their changing bodies, using written prompts to increase self-compassion towards themselves and their post-cancer bodies. The intervention also includes participants practicing self- kindness, common humanity, and mindfulness IG: self-compassion-focused writing CG: unstructured expressive writing and usual care Instructor: self-guided	Web-based	30-min, single-session, online writing Follow-up occurred 1 week, 1 month, and 3 months after writing	Body image distress Body appreciation Psychological distress (depression and anxiety) Self-compassion Appearance investment	MyCB reported significantly less body image distress and greater body appreciation and self-compassion than the control group

IG, ıntervention group; CG, control group; MyCB, My Changed Body

### Findings relating to intervention type

The eight studies were presented by categorizing the type of intervention into four categories. The categories include the mindful self-compassion (MSC), compassionate mind training (CMT), self-compassionate-focused writing, and kindness interventions.

#### Mindful self-compassion

Three studies included in the systematic review implemented MSC interventions [6, 7, 45]. In two studies, the MSC intervention developed by Neff and Germer was applied [6, 7, 28], while in one study, the MSC intervention adapted from the book “Making Friends with Yourself”, developed by Germer, Neff [47] and Bluth [48] was applied [45]. The interventions include applications, compassionate friend meditation, body scan, here and now stone, affectionate breathing meditation, lovingkindness meditation, soften-soothe-allow meditation, gratitude phone photos [45], coping with difficult emotions and negative core beliefs, finding your compassionate inner voice, self-compassionate body scan, foot meditation and self-appreciation [7]. Mindful breathing and movement, present-moment awareness exercises, compassionate imagery, compassionate letter writing, peer sharing, and normalization of experiences, home assignments consisting of daily audio-guided practice and reflective journaling tasks were also among the implementations carried out [6]. The interventions in this group were delivered by instructors who had received professional training in mindful self-compassion [6, 7, 45].

#### Self-compassion-focused writing

The self-compassion-focused writing intervention developed by Leary and colleagues [27] was implemented in the three studies included in the systematic review [25, 31, 46]. In the intervention, patients were encouraged to write about their deepest thoughts and feelings regarding a negative event related to their changing bodies [31, 46]. Patients continued to write using written prompts designed to increase self-compassion towards themselves and their post-cancer bodies. The prompts encouraged participants to practice self-kindness, common humanity, and mindfulness [31, 46]. In the intervention carried out by Mifsud et al., patients were invited to write a compassionate letter to themselves, containing gentle advice to their bodies; to connect with others who may have had similar experiences; and to become mindful of their experiences and reactions [25]. The interventions in this group were self-guided without professional guidance [25, 31, 46].

#### Compassionate mind training

A study included in the systematic review applied a compassionate mind training intervention developed by Gilbert [24, 29]. The intervention included psychoeducation on soothing, acting, and threat systems, calming breathing rhythm exercises, imagery exercises, self-compassion-based expressive writing exercises, compassionate body scans, and loving-kindness meditation [24]. This intervention was self-guided without professional guidance [24].

#### Kindness interventions

A study included in the systematic review implemented kindness interventions [26]. The intervention included acts of kindness to others, acts of kindness to oneself, self-kindness meditation, and daily activity writing [26]. Patients in the kindness to others group were invited to engage in kind, generous, or thoughtful acts towards others, such as paying for another patient’s coffee, writing a thank-you note, or making a cup of tea for their spouse [26]. Patients in the kindness to oneself group were invited to engage in acts of kindness towards themselves, such as going for a lunchtime walk or preparing their favorite meal [26]. Patients in the self-compassion meditation group were instructed to listen to a 5-min guided meditation each week [26]. During each meditation, participants were asked to send themselves compassion and repeat phrases such as “I am peaceful, “I am healthy and strong, and “I am safe” [26]. Only the audio files recorded during self-compassion meditation were recorded by an experienced mindfulness instructor. The other interventions were self-guided [26].

### Findings relating to program characteristics: delivery and duration

All interventions were delivered online. Three studies were conducted using a video conferencing tool [6, 7, 45]. Cisco WebEx [45], Tencent Meetings [7], and Zoom [6] were used as video conferencing tools. Three studies utilized the web based My Changed Body (MyCB) [25, 31, 46]. In one study, MyCB was delivered via both booklets and a web based. 59% of participants chose the booklets [46]. An intervention was carried out via the Compas-Y mobile application, developed in collaboration with patients with cancer and oncology nurses [24]. In one study, self-compassion meditation was offered to patients via Blogger, an online blog-publishing platform. Although it is reported that other interventions were delivered online, the method of delivery is not reported in the study [26].

Online self-compassion interventions were examined in terms of duration. The intervention durations of the eight studies included in the systematic review ranged from 1 week [25, 31, 46] to 8 weeks [24, 45]. It has been found that the least effective interventions are those of shorter duration [25, 31, 46]. It has been found that a 4-week intervention may have no effect or may have detrimental effects on patient outcomes [26]. However, the most effective interventions were found to be those with the longest duration [7, 45]. The 8-week intervention carried out by Austin et al. is an exception to this situation [24]. The duration of the included interventions varied between 5 min [26] and 90 min [45]. It was found that a 90-min intervention had a positive effect on patient outcomes [45], whereas a 5-min intervention could have negative effects on patient outcomes [26].

### Findings relating to cancer type

*Breast cancer*: The effects of interventions on breast cancer patients were evaluated in the seven studies included in the systematic review [6, 7, 24–26, 31, 45]. Five studies included only breast cancer patients [6, 7, 25, 26, 31], while two studies included different types of cancer. Breast cancer was among the cancers included [24, 45]. In the included studies, participants had varied cancer stages. One study included patients diagnosed with stages 0, I, II, or IIIA early-stage breast cancer [26], while two studies included patients with stages 0–IV a broader disease spectrum [6, 7]. Two studies focused on patients with stage I to III breast cancer [25, 31].

*Other cancer*: Two studies included in the systematic review included different types of cancer [24, 45]. Other types of cancer included are colorectal or esophageal [24], thyroid [45], lymphoma [24, 45], leukemia [45], melanoma [24], sarcoma [45], prostate [24], ovarian [24, 45], lung [24, 45], central nervous system [45], and brain cancer [45]. One study included only head and neck cancers [46].

### Findings relating to patient outcomes

Table 2 shows the effects of online self-compassion interventions in patients with cancer. Patient outcomes were categorized into five groups: findings related to “self-compassion and sub-dimension outcomes,” “anxiety, stress and depression outcomes,” “body image outcomes,” “well-being and sub-dimension outcomes,” and “other outcomes.”

**Table 2:** Effects of online self-compassion interventions in patients with cancer

Outcomes categories	Patient outcomes	Statistically significant	Not statistically significant
**Self-compassion and sub-dimensions outcomes**	Self-compassion	Melissant et al., 2021; Mifsud et al., 2021; Campo et al., 2017; Chen et al., 2024	Sherman et al., 2018; Austin et al., 2023
	Self-kindness	Haydon et al., 2023 (significant but unexpected negative impact)	Haydon et al., 2023
	Mindfulness	Campo et al., 2017	
	Self-criticism	Austin et al., 2023	
	Social isolation	Campo et al., 2017	Austin et al., 2023
**Anxiety, stress, and depression outcomes**	Anxiety	Austin et al., 2023; Campo et al., 2017; Mifsud et al., 2021	Sherman et al., 2018
	Stress	Chen et al., 2024	Mifsud et al., 2021; Melissant et al., 2021
	Depression	Campo et al., 2017	Sherman et al., 2018; Mifsud et al., 2021; Haydon et al., 2023; Austin et al., 2023
**Body image outcomes**	Body image distress	Mifsud et al., 2021; Campo et al., 2017; Chen et al., 2024	Sherman et al., 2018; Melissant et al., 2021
	Body image appreciation	Sherman et al., 2018	Melissant et al., 2021; Mifsud et al., 2021
**Well-being and sub-dimensions outcomes**	Well-being	Çalışkan ve Kutlu, 2025	Haydon et al., 2023; Austin et al., 2023
	Hope	Çalışkan ve Kutlu, 2025	
	Take Action	Çalışkan ve Kutlu, 2025	
	Nothingness	Çalışkan ve Kutlu, 2025	
	Regret		Çalışkan ve Kutlu, 2025
**Other outcomes**	Self-acceptance	Chen et al., 2024	
	Posttraumatic growth	Campo et al., 2017	
	Positive affect	Mifsud et al., 2021	
	Problems with social contact		Melissant et al., 2021
	Social support		Haydon et al., 2023
	Resilience		Campo et al., 2017; Austin et al., 2023
	Health-related quality of life		Melissant et al., 2021
	Cognitive coping and emotion regulation strategies		Austin et al., 2023
	Negative affect		Mifsud et al., 2021
	Fears of giving and receiving compassion		Austin et al., 2023
	Sexual problems		Melissant et al., 2021
	Problems with wound healing		Melissant et al., 2021

#### Findings relating to self-compassion and sub-dimensions outcomes

 Six studies evaluated the effect of an online self-compassion intervention on self-compassion levels [7, 24, 25, 31, 45, 46]; four studies found an increase in patients’ self-compassion levels [7, 25, 45, 46], while two studies found no significant difference [24, 31]. A study evaluated self- kindness, the positive sub-dimension of self-compassion [26]. In this study, while the level of self-kindness decreased in the self-kindness meditation, no significant difference was observed in patients’ level of self-kindness in the acts of kindness to others and acts of kindness to oneself interventions [26]. In one study, mindfulness, a positive sub-dimension of self-compassion, was evaluated, and patients’ levels of mindfulness increased [45]. In one study, self-criticism, the negative sub-dimension of self-compassion, was evaluated, and a decrease in patients’ levels of self-criticism was observed [24]. In one study, social isolation, one of the negative subdimensions of self-compassion, was assessed, and a decrease in patients’ levels of social isolation was found [45].

#### Findings relating to anxiety, stress, and depression outcomes

In four studies, patients’ anxiety levels were evaluated [24, 25, 31, 45]; in three of them, anxiety levels decreased [24, 25, 45], while in one study, no significant difference was found [31]. In three studies, patients’ stress levels were evaluated [7, 25, 46], and only one study found that stress levels decreased [7]. In five studies, patients’ depression levels were assessed [24–26, 31, 45], and only in one study had patients’ depression levels decreased [45].

#### Findings relating to body image outcomes

Body image distress was assessed in five studies [7, 25, 31, 45, 46], and body image distress decreased in three studies [7, 25, 45]. Body image appreciation was assessed in three studies [25, 31, 46], and only one study increased patients’ levels of body image appreciation [31].

#### Findings relating to well-being and sub-dimension outcomes

In three studies, patients’ well-being levels were evaluated [6, 24, 26], and only one study increased patients’ well-being levels [6]. In one study, the levels of nothingness, hope, regret, and taking action, which are sub-dimensions of well-being, were assessed. Hope and take action levels increased, while nothingness levels decreased. No significant difference was found in the regret sub-dimension [6].

#### Findings relating to other outcomes

While patients’ self-acceptance [7], post-traumatic growth [45], and positive affect [25] increased, no statistically significant differences were found in levels of problems with social contact [46], social support [26], resilience [24, 45], health-related quality of life [46], cognitive coping and emotion regulation strategies [24], negative affect [25], fear of giving and receiving compassion [24], sexual problems [46], and problems with wound healing [46].

### Sensitivity analysis

A sensitivity analysis was performed to assess the robustness of the findings, including only randomized controlled trials and excluding quasi-experimental designs [6, 7, 25, 26, 31]. The findings remained robustness, indicating that the effect of online self-compassion interventions on self-compassion [7, 25, 31], self-kindness [26], stress [7, 25], depression [25, 26, 31], body image distress [7, 25, 31], body image appreciation [25, 31], well-being [6, 26], hope [6], take action [6], nothingness [6], regret [6], self-acceptance [7], positive affect [25], negative affect [25], and social support [26] levels was maintained after excluding quasi-experimental studies. However, the findings related to the outcomes self-kindness [26], hope [6], take action [6], nothingness [6], regret [6], self-acceptance [7], positive affect [25], negative affect [25], and social support [26] were derived from only a single randomized controlled trial. Additionally, mindfulness [45], self-criticism [24], social isolation [45], posttraumatic growth [45], problems with social contact [46], resilience [24, 45], health-related quality of life [46], cognitive coping and emotion regulation strategies [24], fears of giving and receiving compassion [24], sexual problems [46], problems with wound healing [46] since the outcome variables were only evaluated in studies with a quasi-experimental design, they could not be re-evaluated in the sensitivity analysis. Additionally, sensitivity analyses have shown that the effects on only anxiety not robust due to variability in some study results or loss of statistical significance [25, 31] (Appendix File, Table S2).

### Quality assessment of the included studies

**Fig. 2:**
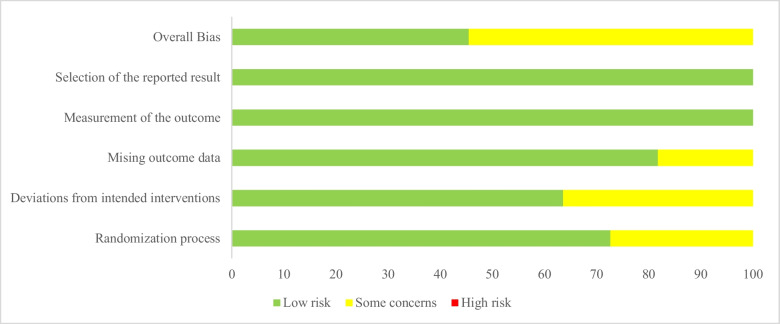
Proportion of bias domains

The risk of bias assessment is presented in Fig. 2 and Table 4 in line with Cochrane criteria. Figure 2 shows the proportion risk of bias for five randomized controlled trials. Table 3 shows the summary of risk of bias of included studies. The randomization process was found to be low risk in four studies [6, 7, 25, 31] and some concerns in one study [26]. One study on deviations from the intended intervention was double-blind [25], and two study was single-blind [6, 31]. Blinding status was not explicitly stated in two of the studies [7, 26]. Therefore, while two trials were found to have a low risk of deviations from the intended intervention [25, 31], two trials were assessed as having some concern due to there being no information about whether participants, carers, and the people administering the interventions to the participants were aware of the intervention assigned to them during the trial [7, 26]. In all studies except one [6], missing outcome data were assessed as low risk [7, 25, 26, 31]. The status of missing outcome data was assessed as participants dropped out of the study or were excluded from the analysis. In randomized controlled trials, intention-to-treat analysis is required, with four studies having an intention-to-treat (ITT) analysis [7, 25, 26, 31]. Although there were 10 missing data points in one study, it was evaluated as “some concerns” because the appropriate analysis was not performed [6]. In all included studies, the risk of bias in the measurement of the outcome and the risk of bias in the selection of the reported outcome were found to be low risk [6, 7, 25, 26, 31] (Fig. 2) (Table 3). According to the JBI tool assessment presented in Table 4, all quasi-experimental studies were assessed as low risk in terms of temporal precedence bias [24, 45, 46]. In all studies, follow-up was completed, differences between groups were analyzed, results were measured in the same way, and appropriate analysis was used [24, 45, 46]. The major limitation of quasi-experimental studies is the lack of a control group [24, 45, 46]. The studies showed varying levels of bias regarding confounding factors, intervention/exposure administration, outcome assessment, detection, measurement, and measurement tools (Table 4).

**Table 3:**
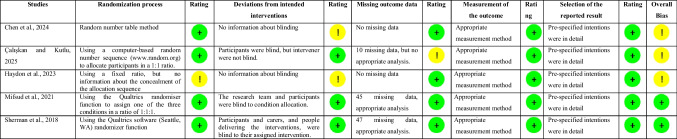
The summary of risk of bias of included studies

**Table 4:** JBI critical appraisal tool for quasi-experimental studies

Criteria*	*Austin *et al*., 2023*	*Campo *et al*., 2017*	*Melissant *et al*., 2021*
1. Is it clear in the study what is the ‘cause’ and what is the ‘effect’ (i.e. there is no confusion about which variable comes first)?	Yes	Yes	Yes
2. Was there a control group?	No	No	No
3. Were the participants included in any comparisons similar?	Unclear	Yes	Yes
4. Were the participants included in any comparisons receiving similar treatment/care, other than the exposure or intervention of interest?	Yes	Unclear	Unclear
5. Were there multiple measurements of the outcome both pre and post the intervention/exposure?	Yes	No	Yes
6. Was follow up complete and if not, were differences between groups in terms of their follow up adequately described and analyzed?	Yes	Yes	Yes
7. Were the outcomes of participants included in any comparisons measured in the same way?	Yes	Yes	Yes
8. Were outcomes measured in a reliable way?	No	Yes	Unclear
9. Was appropriate statistical analysis used?	Yes	Yes	Yes

^*^Evaluations were carried out according to Yes/No/Unclear/N/A

## Discussion

In this systematic review, which aimed to investigate the effectiveness of online self-compassion-based interventions and their impact on cancer patients’ outcomes, eight studies were analyzed, and the studies were conducted in Australia, the Netherlands, the USA, China, and Türkiye. The review showed that current interventions include mindful self-compassion [6, 7, 45], self-compassion-focused writing [25, 31, 46], compassionate mind training [24], and kindness interventions [26]. Interventions were delivered via the video conferencing tool [6, 7, 45], web based [25, 31, 46], mobile application [24], and blog publishing platform [26]. Online self-compassion interventions have mostly been implemented for patients with breast cancer [6, 7, 24–26, 31, 45]. The interventions were evaluated in terms of patient outcomes, including self-compassion [7, 24, 25, 31, 45, 46] and its subdimensions [24, 26, 45], anxiety [24, 25, 31, 45], stress [7, 25, 46] and depression [24–26, 31, 45], body image [7, 25, 31, 45, 46], well-being [6, 24, 26], and its subdimensions [6], and other outcomes [7, 24–26, 45, 46].

According to the findings of this study, online self-compassion interventions increased cancer patients’ levels of self-compassion [7, 25, 45, 46] and mindfulness [45], the positive sub-dimension of self-compassion. Furthermore, the interventions reduced levels of self-criticism [24] and social isolation [45], the negative sub-dimensions of self-compassion. These findings show that self-compassion is an accessible and can be learned quality in cancer patients [49, 50]. Self-compassion-based interventions can contribute to increased self-compassion by strengthening the sense of common humanity and self-acceptance in uncontrollable situations [51] and by enhancing emotion regulation skills [21]. In addition, exposure to interventions on a regular and repetitive basis can lead to permanent effects on self-compassion [25]. Previous research results in the field also support our systematic review findings [52, 53]. On the other hand, there is also two study in which online self-compassion interventions did not affect patients’ self-compassion levels [24, 31]. The reasons for this include the fact that the participants were members of a pre-existing support network [31], ceiling effects [24], the intervention was limited to a single session [31], there was no long-term follow-up [31], and self-compassion was measured with a published but unvalidated measurement tool [31]. Additionally, it has been found that kindness interventions do not affect patients’ self-kindness levels and may have negative effects. Kindness-based online interventions may not deliver the expected benefits and may even lead to a decrease in self-kindness levels, as they do not include guided support, psychoeducation, peer sharing, and instructor support. In order to improve the risk-benefit analysis, attention should also be given to the potential risks of interventions. Healthcare professionals can utilise mindful self-compassion [7, 45] and self-compassion-focused writing interventions [25, 46] in the clinic to increase patients’ levels of self-compassion. Although the effects of these interventions have been evaluated mostly on patients with breast cancer [7, 25, 45], they have also been effective in head and neck cancer [46], colorectal, esophagus, lymphoma, leukemia, thyroid, melanoma, sarcoma, ovarian, prostate, central nervous system, lung, and brain cancers [24, 45].

Our findings from this systematic review indicate that online self-compassion interventions decrease anxiety [24, 25, 45], stress [7], and depression [45] levels in patients with cancer. These findings show that online self-compassion interventions applied can be used as a supportive tool to reduce anxiety, stress and depression levels of patients. Increased self-compassion in patients with cancer is associated with decreased anxiety, stress, and depression, and this relationship is supported by previous observational studies [8, 54]. A meta-analysis examining the relationship between self-compassion and psychopathology has revealed a moderate-to-strong negative relationship between self-compassion and symptoms of anxiety, depression, and stress [49]. Self-compassion can reduce anxiety, stress, and depressive symptoms by improving emotional regulation and coping and reducing repetitive negative thoughts such as worry and rumination [21]. These results also support other research findings in the literature [17, 52]. However, there are also studies in which interventions did not affect the anxiety [31], stress [25], psychological distress [46], and depression [24–26, 31] symptoms of patients with cancer. The reasons for this include newly diagnosed patients [24], the intervention being implemented in a single session [25, 46] and lack of follow-up assessment after the intervention [26], reduced mental health support due to the COVID-19 pandemic, and small sample size [25]. Nurses can utilise compassionate mind training [24], mindful self-compassion [7, 45], and self-compassion-focused writing [25] interventions to reduce patients’ levels of anxiety, stress, and depression. Although the effectiveness of these interventions has been studied mostly in patients with breast cancer [7, 25], they can also be carried out in patients with lymphoma, leukemia, thyroid, colorectal or esophageal cancer, sarcoma, ovarian cancer, central nervous system cancer, lung cancer, brain cancer, prostate cancer, or melanoma [24, 45].

Findings from this systematic review indicate that online self-compassion interventions are effective in increasing body image appreciation [31] levels in cancer patients and decreasing body image distress [7, 25, 45]. Studies addressing body image distress have mostly focused on patients with breast cancer [7, 25, 31]. In this context, body image stress is not merely an aesthetic concern; it is a psychosocial stressor related to identity, self-esteem, and perceptions of femininity [55]. In the literature, self-compassion is reported to be a protective factor against body image distress [31]. Self-compassion-based interventions may alleviate body image distress by reducing self-criticism towards cancer-related physical changes and promoting acceptance of the body after treatment [25]. Patients with cancer who have high levels of self-compassion, when faced with these changes in their body appearance and functional dimensions, can see them as part of the human experience and accept such changes in their bodies more affectionately rather than resorting to being self-critical, thus reducing body image stress and improving their quality of life [31]. Therefore, practicing self-compassion intervention can contribute to a more positive body image and reduce body image distress. However, studies also show that self-compassion interventions do not affect body image distress [31, 46] and appreciation [25, 46]. The reasons for this include the intervention being implemented in a single session [25, 31, 46] and the lack of long-term follow-up of the intervention [25, 31], low body image stress at the beginning, differences in symptoms between patients with breast cancer [46], and single-session application [31]. Future studies are recommended to evaluate the long-term effects of interventions and conduct long-term follow-ups. Nurses can utilise mindful self-compassion and self-compassion-focused writing interventions to reduce body image distress and increase body image appreciation. Although the effects of these interventions have been studied mostly on individuals with breast cancer [7, 25, 31], they have also been effective in patients with lymphoma, leukemia, thyroid, sarcoma, ovarian, central nervous system, lung, and brain cancer [45].

The findings of this systematic review indicate that online self-compassion interventions may increase cancer patients’ well-being levels [6], hope and take action sub-dimensions of well-being [6], and reduce the nothingness sub-dimension of well-being [6]. However, there are also two studies showing that interventions do not affect well-being [24, 26]. A systematic review evaluating the effects of online self-compassion interventions indicates that these interventions improve well-being by increasing self-compassion [18]. As self-compassion increases, individuals move away from threat-based emotional responses towards greater emotional security, acceptance, and self-soothing [18]. This process supports improvements in mixed well-being outcomes that combine hedonistic and eudaimonic dimensions, with some benefits emerging gradually over time [18]. The reason why the two interventions were ineffective may be due to the fact that their long-term effects have not been studied [26] and that they were insufficient in increasing self-compassion [24]. Nurses can use mindful self-compassion interventions to improve patients’ well-being. These interventions were effective only in patients with breast cancer [6].

Sensitivity analysis, conducted by including only randomized controlled trials and excluding quasi-experimental designs to assess the robustness of the findings, found that online self-compassion interventions maintained a robustness effect on self-compassion [7, 25, 31], self-kindness [26], stress [7, 25], depression [25, 26, 31], body image distress [7, 25, 31], body image appreciation [25, 31], well-being [6, 26], hope [6], take action [6], nothingness [6], regret [6], self-acceptance [7], positive affect [25], social support [26], and negative affect [25] but their effects on anxiety [25, 31] were not robust. When interpreting the effects of interventions on anxiety, the results of sensitivity analysis should be considered. However, since the findings regarding self-kindness [26], hope [6], take action [6], nothingness [6], regret [6], self-acceptance [7], positive affect [25], negative affect [25], and social support [26] were derived from only one randomized controlled trial, the results should be interpreted with caution. Since mindfulness [45], self-criticism [24], social isolation [45], posttraumatic growth [45], problems with social contact [46], resilience [24, 45], health-related quality of life [46], cognitive coping and emotion regulation strategies [24], fears of giving and receiving compassion [24], sexual problems [46], problems with wound healing [46] were obtained from non-randomized controlled studies therefore, we recommend that randomized controlled trials be planned to investigate these outcomes. In addition to this, future studies are recommended to conduct long-term follow-up studies on online self-compassion interventions for patients with cancer. Especially, follow-up assessments lasting at least 6 months can help determine whether improvements in key outcomes such as self-compassion and body image distress are sustained over time. Furthermore, future studies should document potential adverse events associated with the intervention to improve a more comprehensive risk-benefit analysis framework. Such designs will contribute to stronger evidence base regarding the long-term efficacy and safety of online self-compassion interventions.

### Limitations

This systematic review has some limitations. First, due to the limited number of studies, different study types, such as quasi-experimental studies and randomized controlled designs, as well as heterogeneous self-compassion interventions such as mindful self-compassion, self-compassion-focused writing, compassion mind training, and kindness interventions have been included. This may reduce the reliability of the results. Furthermore, a sensitivity analysis that included only randomized controlled trials concluded that the findings regarding anxiety were not robust. Results derived from a single randomized controlled trial, as well as those from a quasi-experimental study design despite being statistically significant should be interpreted with caution. Secondly, although the systematic review examined the effects of interventions in different patients with cancer, the findings regarding the effects of interventions were mostly obtained from breast cancer patients. Therefore, it is recommended that the effectiveness of the interventions be examined in various patients with cancer. Many of the reviewed studies had small sample sizes, which also constitutes another limitation. Another limitation of the reviewed studies is that the interventions were applied in a single session, the follow-up was short, and they were single-center studies. The long-term effects of interventions are unknown due to the short follow-up periods of the included studies, and there is a lack of data on the safety of long-term interventions. There is limited information on the long-term effects and side effects of interventions in the included studies. Only studies published in English have been included in this systematic review. This situation may have led to language bias. Finally, a meta-analysis was not performed due to the different outcomes.

## Conclusions

This systematic review evaluated the effects of online self-compassion-based interventions on patient outcomes in patients with cancer. The results of this systematic review showed that online self-compassion-based interventions can have many positive effects, such as reducing levels of self-criticism, social isolation, anxiety, stress, depression, body image distress, and increasing levels of self-compassion, mindfulness, body image appreciation, well-being, self-acceptance, posttraumatic growth, and positive affect. However, some of the results were obtained from a single randomized controlled trial or from studies with only a quasi-experimental design; therefore, we recommend that these results be interpreted with caution. In addition, the results of the sensitivity analysis should be taken into account when interpreting the results. We recommend that future studies investigate the effects and potential side effects of online self-compassion interventions more comprehensively using larger sample sizes, longer intervention durations, follow-up periods of at least 6 months, and multicenter studies. Furthermore, future research should prioritize high-quality randomized controlled trials that report methodological procedures transparently.

## Supplementary Information

Below is the link to the electronic supplementary material.

ESM 1 (DOCX 18.7 KB)

## Data Availability

The data that support the findings of this study are available from the corresponding author upon reasonable request.

## References

[1] Fitzmaurice C, Abate D, Abbasi N, Abbastabar H, Abd-Allah F, Abdel-Rahman O et al (2019) Global, regional, and national cancer incidence, mortality, years of life lost, years lived with disability, and disability-adjusted life-years for 29 cancer groups, 1990 to 2017: a systematic analysis for the Global burden of disease study. JAMA Oncol 5(12):1749–68. 10.1001/jamaoncol.2019.2996 31560378 10.1001/jamaoncol.2019.2996 PMC6777271

[2] GLOBOCAN (2022) Cancer fact sheets. Lyon, France: International Agency for Research on Cancer. https://gco.iarc.fr/today/fact-sheets-cancers. Accessed 20 May 2025.

[3] Harris CS, Kober KM, Conley YP, Dhruva AA, Hammer MJ, Miaskowski CA (2022) Symptom clusters in patients receiving chemotherapy: a systematic review. BMJ Support Palliat Care 12(1):10–21. 10.1136/bmjspcare-2021-003325 34921000 10.1136/bmjspcare-2021-003325 PMC8857036

[4] Neff K (2003) The development and validation of a scale to measure self-compassion. Self Identity 2:223–250. 10.1080/15298860309027

[5] Neff K (2003) Self-compassion: an alternative conceptualization of a healthy attitude toward oneself. Self Identity 2(2):85–101. 10.1080/15298860309032

[6] Çalışkan BB, Kutlu FY (2025) Mindfulness-based self-compassion to enhance ontological well-being in breast cancer survivors: a randomized controlled trial. Eur J Oncol Nurs. 10.1016/j.ejon.2025.102983 41061577 10.1016/j.ejon.2025.102983

[7] Chen Y, Liu R, Xiao J, Wang Y, Yang Y, Fan H et al (2024) Effects of online mindful self-compassion intervention on negative body image in breast cancer patients: a randomized controlled trail. Eur J Oncol Nurs 72:102664. 10.1016/j.ejon.2024.102664 39059197 10.1016/j.ejon.2024.102664

[8] Wei L, Xie J, Wu L, Yao J, Zhu L, Liu A (2023) Profiles of self-compassion and psychological outcomes in cancer patients. Psycho-Oncol 32(1):25–33. 10.1002/pon.5931 10.1002/pon.5931 35334138

[9] Çetiner E, Turan Kavradım S (2025) Relationship between fatigue and self-compassion and associated factors in patients with hematological cancer: a cross-sectional study. Support Care Cancer 33(12):1039. 10.1007/s00520-025-10006-w 41214375 10.1007/s00520-025-10006-w

[10] Zhu F, Zhang W, Liu C, Qiang W, Lu Q (2023) Association of self-compassion and body image disturbance among young breast cancer patients: mediating effect of body surveillance and body shame. Asia-Pac J Oncol Nurs 10(4):100199. 10.1016/j.apjon.2023.100199 36923469 10.1016/j.apjon.2023.100199 PMC10009058

[11] Houston EE, Brown L, Jones KM, Amonoo HL, Bryant C (2023) Does self-compassion explain variance in sleep quality in women experiencing hot flushes? Maturitas 172:39–45. 10.1016/j.maturitas.2023.04.003 37099982 10.1016/j.maturitas.2023.04.003

[12] Khalili N, Bahrami M, Ashouri E (2021) Self-compassion and adherence to treatment in patients with cancer. Iran J Nurs Midwifery Res 26(5):406–410. 10.4103/ijnmr.IJNMR_174_20 34703778 10.4103/ijnmr.IJNMR_174_20 PMC8491826

[13] Masoumi S, Amiri M, Yousefi Afrashteh M (2022) Self-compassion: the factor that explains a relationship between perceived social support and emotional self-regulation in psychological well-being of breast cancer survivors. Iran J Psychiatry 17(3):341–349. 10.18502/ijps.v17i3.9734 36474692 10.18502/ijps.v17i3.9734 PMC9699804

[14] Garcia ACM, Junior JBC, Sarto KK, da Silva Marcelo CA, das Chagas Paiva EM, Nogueira DA et al (2021) Quality of life, self-compassion and mindfulness in cancer patients undergoing chemotherapy: a cross-sectional study. Eur J Oncol Nurs 51:101924. 10.1016/j.ejon.2021.101924 33610930 10.1016/j.ejon.2021.101924

[15] Brooker J, Julian J, Millar J, Prince HM, Kenealy M, Herbert K et al (2020) A feasibility and acceptability study of an adaptation of the Mindful Self-Compassion program for adult cancer patients. Palliat Support Care 18(2):130–40. 10.1017/S1478951519000737 31595861 10.1017/S1478951519000737

[16] Hoffman C, Baker B (2023) Effects of mindful self-compassion program on psychological well-being and levels of compassion in people affected by breast cancer. Altern Ther Health Med 29:36–41 35648691

[17] Austin J, Drossaert CHC, Schroevers MJ, Sanderman R, Kirby JN, Bohlmeijer ET (2021) Compassion-based interventions for people with long-term physical conditions: a mixed methods systematic review. Psychol Health 36(1):16–42. 10.1080/08870446.2019.1699090 32116052 10.1080/08870446.2019.1699090

[18] Randhawa AK, Vella-Brodrick DA (2025) Online self-compassion interventions and wellbeing outcomes: a systematic review of RCTs. Mindfulness. 10.1007/s12671-025-02606-8

[19] Finlay-Jones A, Boyes M, Perry Y, Sirois F, Lee R, Rees C (2020) Online self-compassion training to improve the wellbeing of youth with chronic medical conditions: protocol for a randomised control trial. BMC Public Health 20(1):106. 10.1186/s12889-020-8226-7 31992269 10.1186/s12889-020-8226-7 PMC6986046

[20] Ramsenthaler C, Kane P, Gao W, Siegert RJ, Edmonds PM, Schey SA et al (2016) Prevalence of symptoms in patients with multiple myeloma: a systematic review and meta-analysis. Eur J Haematol 97(5):416–29. 10.1111/ejh.12790 27528496 10.1111/ejh.12790

[21] Wang J, Drossaert CH, Knevel M, Chen L, Bohlmeijer ET, Schroevers MJ (2025) The mechanisms underlying the relationship between self-compassion and psychological outcomes in adult populations: a systematic review. Stress Health 41(4):e70090. 10.1002/smi.70090 40719190 10.1002/smi.70090 PMC12302336

[22] Li W, Zhang X, Yuan M, Hu J, Li S (2023) Factors associated with self-compassion in Chinese oesophageal cancer patients undergoing oesophagectomy: based on self-determination theory. Curr Psychol 43:1–13. 10.1007/s12144-023-05497-x

[23] Brunet J, Sabiston CM, Burke S (2013) Surviving breast cancer: women’s experiences with their changed bodies. Body Image 10(3):344–351. 10.1016/j.bodyim.2013.02.002 23490552 10.1016/j.bodyim.2013.02.002

[24] Austin J, Schroevers MJ, Van Dijk J, Sanderman R, Børøsund E, Wymenga AMN et al (2023) Compas-Y: a mixed methods pilot evaluation of a mobile self-compassion training for people with newly diagnosed cancer. Digit Health 9:20552076231205272. 10.1177/20552076231205272 37868157 10.1177/20552076231205272 PMC10588427

[25] Mifsud A, Pehlivan MJ, Fam P, O’Grady M, van Steensel A, Elder E et al (2021) Feasibility and pilot study of a brief self-compassion intervention addressing body image distress in breast cancer survivors. Health Psychol Behav Med 9(1):498–526. 10.1080/21642850.2021.1929236 34104572 10.1080/21642850.2021.1929236 PMC8158280

[26] Haydon MD, Walsh LC, Fritz MM, Rahal D, Lyubomirsky S, Bower JE (2023) Kindness interventions for early-stage breast cancer survivors: an online, pilot randomized controlled trial. J Posit Psychol 18(5):743–754. 10.1080/17439760.2022.2093786

[27] Leary MR, Tate EB, Adams CE, Batts Allen A, Hancock J (2007) Self-compassion and reactions to unpleasant self-relevant events: the implications of treating oneself kindly. J Pers Soc Psychol 92(5):887. 10.1037/0022-3514.92.5.887 17484611 10.1037/0022-3514.92.5.887

[28] Neff KD, Germer CK (2013) A pilot study and randomized controlled trial of the mindful self‐compassion program. J Clin Psychol 69(1):28–44. 10.1002/jclp.21923 23070875 10.1002/jclp.21923

[29] Gilbert P (2014) The origins and nature of compassion focused therapy. Br J Clin Psychol 53(1):6–41. 10.1111/bjc.12043 24588760 10.1111/bjc.12043

[30] Torrijos‐Zarcero M, Mediavilla R, Rodríguez‐Vega B, Del Río‐Diéguez M, López‐Álvarez I, Rocamora‐González C et al (2021) Mindful self‐compassion program for chronic pain patients: a randomized controlled trial. Eur J Pain 25(4):930–44. 10.1002/ejp.1734 33471404 10.1002/ejp.1734

[31] Sherman KA, Przezdziecki A, Alcorso J, Kilby CJ, Elder E, Boyages J et al (2018) Reducing body image-related distress in women with breast cancer using a structured online writing exercise: results from the my changed body randomized controlled trial. J Clin Oncol 36(19):1930–40. 10.1200/JCO.2017.76.3318 29688834 10.1200/JCO.2017.76.3318

[32] Przezdziecki A, Sherman KA, Baillie A, Taylor A, Foley E, Stalgis-Bilinski K (2013) My changed body: breast cancer, body image, distress and self-compassion. Psycho-Oncol 22(8):1872–1879. 10.1002/pon.3230 10.1002/pon.3230 23203842

[33] Kearney K, Hicks RE (2017) Self-compassion and breast cancer in 23 cancer respondents: is the way you relate to yourself a factor in disease onset and progress? Psychol. 10.4236/psych.2017.81002

[34] Cillessen L, Johannsen M, Speckens AEM, Zachariae R (2019) Mindfulness-based interventions for psychological and physical health outcomes in cancer patients and survivors: a systematic review and meta-analysis of randomized controlled trials. Psychooncology 28(12):2257–2269. 10.1002/pon.5214 31464026 10.1002/pon.5214 PMC6916350

[35] Jassim GA, Whitford DL, Hickey A, Carter B (2015) Psychological interventions for women with non-metastatic breast cancer. Cochrane Database Syst Rev 5:Cd008729. 10.1002/14651858.CD008729.pub3 10.1002/14651858.CD008729.pub3 PMC9832339 36628983

[36] Linardon J (2020) Can acceptance, mindfulness, and self-compassion be learned by smartphone apps? A systematic and meta-analytic review of randomized controlled trials. Behav Ther 51(4):646–658. 10.1016/j.beth.2019.10.002 32586436 10.1016/j.beth.2019.10.002

[37] Bailey NW, Comte W, Chambers R, Bartlett L, Connaughton S, Hassed C (2023) Participation in online mindfulness was associated with improved self-compassion and prosocial attitudes. Mindfulness 14(7):1735–1750. 10.1007/s12671-023-02168-7

[38] Bégin C, Berthod J, Martinez LZ, Truchon M (2022) Use of mobile apps and online programs of mindfulness and self-compassion training in workers: a scoping review. J Technol Behav Sci 7(4):477–515. 10.1007/s41347-022-00267-1 36091081 10.1007/s41347-022-00267-1 PMC9444703

[39] Nadeau MM, Caporale-Berkowitz NA, Rochlen AB (2021) Improving women’s self-compassion through an online program: a randomized controlled trial. J Counsel Dev 99(1):47–59. 10.1002/jcad.12353

[40] Mojtabai R, Olfson M, Sampson NA, Jin R, Druss B, Wang PS et al (2011) Barriers to mental health treatment: results from the National Comorbidity Survey Replication. Psychol Med 41(8):1751–61. 10.1017/S0033291710002291 21134315 10.1017/S0033291710002291 PMC3128692

[41] Zhao D, Lustria MLA, Hendrickse J (2017) Systematic review of the information and communication technology features of web-and mobile-based psychoeducational interventions for depression. Patient Educ Couns 100(6):1049–1072. 10.1016/j.pec.2017.01.004 10.1016/j.pec.2017.01.004 28126383

[42] Linardon J, Cuijpers P, Carlbring P, Messer M, Fuller-Tyszkiewicz M (2019) The efficacy of app-supported smartphone interventions for mental health problems: a meta-analysis of randomized controlled trials. World Psychiatry 18(3):325–336. 10.1002/wps.20673 10.1002/wps.20673 PMC6732686 31496095

[43] Higgins JPT, Thomas, J., Chandler, J., Cumpston, M., Li, T., Page, M.J. and Welch, V.A (2021) Cochrane handbook for systematic reviews of interventions version 6.2 (updated February 2021). Available from: www.training.cochrane.org/handbook

[44] Moola S, Munn Z, Tufanaru C, Aromataris E, Sears K, Sfetc R, et al (2020) Chapter 7: Systematic reviews of etiology and risk. 2. 10.1097/XEB.0000000000000064

[45] Campo RA, Bluth K, Santacroce SJ, Knapik S, Tan J, Gold S et al (2017) A mindful self-compassion videoconference intervention for nationally recruited posttreatment young adult cancer survivors: feasibility, acceptability, and psychosocial outcomes. Support Care Cancer 25(6):1759–68. 10.1007/s00520-017-3586-y 28105523 10.1007/s00520-017-3586-y

[46] Melissant HC, Jansen F, Eerenstein SEJ, Cuijpers P, Lissenberg-Witte BI, Sherman KA et al (2021) A structured expressive writing activity targeting body image-related distress among head and neck cancer survivors: who do we reach and what are the effects? Support Care Cancer 29(10):5763–76. 10.1007/s00520-021-06114-y 33738593 10.1007/s00520-021-06114-y PMC8410700

[47] Germer CK, Neff KD (2013) Self-compassion in clinical practice. J Clin Psychol 69(8):856–867. 10.1002/jclp.22021 23775511 10.1002/jclp.22021

[48] Bluth K, Gaylord SA, Campo RA, Mullarkey MC, Hobbs L (2016) Making friends with yourself: a mixed methods pilot study of a mindful self-compassion program for adolescents. Mindfulness 7(2):479–492. 10.1007/s12671-015-0476-6 27110301 10.1007/s12671-015-0476-6 PMC4838201

[49] MacBeth A, Gumley A (2012) Exploring compassion: a meta-analysis of the association between self-compassion and psychopathology. Clin Psychol Rev 32(6):545–552. 10.1016/j.cpr.2012.06.003 22796446 10.1016/j.cpr.2012.06.003

[50] Marsh IC, Chan SWY, MacBeth A (2018) Self-compassion and psychological distress in adolescents-a meta-analysis. Mindfulness (N Y) 9(4):1011–1027. 10.1007/s12671-017-0850-7 30100930 10.1007/s12671-017-0850-7 PMC6061226

[51] Seekis V, Bradley GL, Duffy AL (2020) Does a facebook-enhanced mindful self-compassion intervention improve body image? An evaluation study. Body Image 34:259–269. 10.1016/j.bodyim.2020.07.006 32717627 10.1016/j.bodyim.2020.07.006

[52] Ferrari M, Hunt C, Harrysunker A, Abbott MJ, Beath AP, Einstein DA (2019) Self-compassion interventions and psychosocial outcomes: a meta-analysis of RCTs. Mindfulness 10(8):1455–1473. 10.1007/s12671-019-01134-6

[53] Wilson AC, Mackintosh K, Power K, Chan SWY (2019) Effectiveness of self-compassion related therapies: a systematic review and meta-analysis. Mindfulness 10(6):979–995. 10.1007/s12671-018-1037-6

[54] Zhu L, Wang J, Liu S, Xie H, Hu Y, Yao J et al (2020) Self-compassion and symptoms of depression and anxiety in Chinese cancer patients: the mediating role of illness perceptions. Mindfulness 11(10):2386–96. 10.1007/s12671-020-01455-x

[55] Ahn J, Suh EE (2023) Body image alteration in women with breast cancer: a concept analysis using an evolutionary method. Asia Pac J Oncol Nurs 10(5):100214. 10.1016/j.apjon.2023.100214 37213808 10.1016/j.apjon.2023.100214 PMC10199402

