# Supplementary material for: Online self-compassion-based interventions on patient outcomes in patients with cancer: a systematic review
Source: Support Care Cancer. 2026 Apr 23;34(5):457. doi: 10.1007/s00520-026-10660-8 (PMC13102871; doi:10.1007/s00520-026-10660-8)
Supplement: Supplementary file 1 — (DOCX 18.7 KB) [file 520_2026_10660_MOESM1_ESM.docx]

**Table S1.** Search strategy

| **Population (P)** | tumor OR neoplasm OR neoplasia OR cancer OR “malignant neoplasm” OR malignancy OR oncology OR carcinoma OR chemotherapy OR “cancer survivor” OR “hematologic neoplasm” OR “hematological malignancy” OR “hematopoietic neoplasm” OR hematopoietic OR hematology |
| --- | --- |
| **Intervention (I^1^)** | “self-compassion” OR “self compassion” OR “mindful self-compassion” OR “loving kindness” OR “self-kindness” OR “self-forgiveness” OR “self-compassion training” OR “compassion-focused therapy” OR “compassionate mind” OR “self-compassion meditation” |
| **Intervention (I^2^)** | internet OR web OR online OR videoconference OR e-health OR ehealth OR web-site OR teleconference OR “internet-delivered” OR “web-based guided” OR m-health OR “mobile health” OR “mobile phone” OR tele-health OR “smart phone” OR “smartphone app” OR “cell phone” OR “mobile app” OR “mobile-based” OR “tablet based” OR e-intervention OR e-technology OR “mobile application” OR “mobile device” OR “web app” OR “phone app” OR “electronic application” OR computer |
| **Outcomes (O)** | **-** |
| **Study Design (S)** | “randomized controlled trial” OR “all random” OR control OR “clinical trial” OR “clinical trials” OR “controlled study” OR experiment* OR trial OR “follow up” OR “mixed method” |

**Table S2.** Outcomes of sensitivity analysis including only randomized controlled trials

| **Outcomes Categories** | **Patient outcomes** | **Statistically significant** | **Not statistically significant** |
| --- | --- | --- | --- |
| **Self-compassion and sub-dimensions outcomes** | Self-compassion | Mifsud et al., 2021; Chen et al., 2024 | Sherman et al., 2018 |
|  | Self-kindness | Haydon et al., 2023 (significant but unexpected negative impact) | Haydon et al., 2023 |
| **Anxiety, stress, and depression outcomes** | Anxiety | Mifsud et al., 2021 | Sherman et al., 2018 |
|  | Stress | Chen et al., 2024 | Mifsud et al., 2021 |
|  | Depression |  | Sherman et al., 2018; Mifsud et al., 2021; Haydon et al., 2023 |
| **Body image outcomes** | Body image distress | Mifsud et al., 2021; Chen et al., 2024 | Sherman et al., 2018 |
|  | Body image appreciation | Sherman et al., 2018 | Mifsud et al., 2021 |
| **Well-being and sub-dimensions outcomes** | Well-being | Çalışkan ve Kutlu, 2025 | Haydon et al., 2023 |
|  | Hope | Çalışkan ve Kutlu, 2025 |  |
|  | Take Action | Çalışkan ve Kutlu, 2025 |  |
|  | Nothingness | Çalışkan ve Kutlu, 2025 |  |
|  | Regret |  | Çalışkan ve Kutlu, 2025 |
| **Other outcomes** | Self-acceptance | Chen et al., 2024 |  |
|  | Positive affect | Mifsud et al., 2021 |  |
|  | Social support |  | Haydon et al., 2023 |
|  | Negative affect |  | Mifsud et al., 2021 |
